# Methodological constraints in interpreting serum paraoxonase-1 activity measurements: an example from a study in HIV-infected patients

**DOI:** 10.1186/1476-511X-9-32

**Published:** 2010-03-25

**Authors:** Sandra Parra, Judit Marsillach, Gerard Aragonès, Anna Rull, Raúl Beltrán-Debón, Carlos Alonso-Villaverde, Jorge Joven, Jordi Camps

**Affiliations:** 1 Centre de Recerca Biomèdica, Hospital Universitari de Sant Joan, Institut d'Investigació Sanitària Pere Virgili, Reus, Catalunya, Spain

## Abstract

**Background:**

Paraoxonase-1 (PON1) is an antioxidant enzyme that attenuates the production of the monocyte chemoattractant protein-1 (MCP-1) *in vitro*. Although oxidation and inflammation are closely related processes, the association between PON1 and MCP-1 has not been completely characterised due, probably, to that the current use of synthetic substrates for PON1 measurement limits the interpretation of the data. In the present study, we explored the relationships between the circulating levels of PON1 and MCP-1 in human immunodeficiency virus-infected patients in relation to the multifunctional capabilities of PON1.

**Methods:**

We measured selected variables in 227 patients and in a control group of 409 participants. Serum PON1 esterase and lactonase activities were measured as the rates of hydrolysis of paraoxon and of 5-(thiobutyl)-butyrolactone, respectively. Oxidised LDL and MCP-1 concentrations were determined by enzyme-linked immunosorbent assay. High-density lipoproteins cholesterol, apolipoprotein A-I, and C-reactive protein concentrations were measured by standard automated methods.

**Results:**

There were significant relationships between PON1 activity and several indices of oxidation and inflammation in control subjects and in infected patients. However, these relationships varied not only with disease status but also on the type of substrate used for PON1 measurement.

**Conclusion:**

The present study is a cautionary tale highlighting that results of clinical studies on PON1 may vary depending on the methods used as well as the disease studied. Until more specific methods using physiologically-akin substrates are developed for PON1 measurement, we suggest the simultaneous employment of at least two different substrates in order to improve the reliability of the results obtained.

## Background

Paraoxonase-1 (PON1) is an enzyme with esterase and lactonase activities found in the circulation bound to high-density lipoproteins (HDL). Research into PON1 has increased exponentially over the past few years because many studies associate this enzyme with inflammation and cardiovascular disease. The physiological substrates of PON1 have not been completely delineated, but *in vitro *studies suggest that a key function is to degrade oxidised phospholipids in low-density lipoproteins (LDL) and HDL and, as such, has an antioxidant role [1]. PON1 attenuates the production of the monocyte chemoattractant protein-1 (MCP-1) in cultured endothelial cells. MCP-1 is a pro-inflammatory chemokine involved in the initial steps in the formation of the atheromatous plaque [2]. Previous studies from our group have shown that HIV-infected patients have decreased serum PON1 activities and increased plasma MCP-1 concentrations, and that certain genetic polymorphisms of both molecules are associated with the presence of sub-clinical atherosclerosis [3-5]. Although oxidation and inflammation are closely related processes, the association between PON1 and MCP-1 has not been completely characterised. This is probably due, at least in part, to the limitations of the current methods of measuring serum PON1 activity i.e. the current use of synthetic substrates limits the interpretation of the data since it is not clear to what extent these measured activities reflect the real endogenous physiological activity of the enzyme.

The present study was aimed at extending our observations on the relationships between the circulating levels of PON1 and MCP-1 in HIV-infected patients. Using two assay methods for the different enzyme activities (esterase and lactonase), we sought to evaluate possible discrepancies relating to the analytical methods (different substrates) employed.

## Methods

### Subjects

We studied 227 HIV-infected patients and 409 healthy volunteers. The exclusion criteria for the patients were age ≤ 18 years, and having an AIDS-related disease. The clinical characteristics of these patients have been previously published [3]. The study was approved by the Ethics Committee of *Hospital Universitari de Sant Joan de Reus (Catalonia)*.

### Biochemical analyses

Serum PON1 esterase activity was measured as the hydrolysis of the substrate paraoxon [6], and the lactonase activity was measured as the hydrolysis of the substrate 5-(thiobutyl)-butyrolactone (TBBL) [7]. HDL-cholesterol and apolipoprotein A-I were analysed by standard methods (Beckman-Coulter, Fullerton, CA, USA), oxidised LDL and MCP-1 by enzyme-linked immunosorbent assay (Mercodia, Uppsala, Sweden, and Prepotech, London, UK, respectively), and C-reactive protein (CRP) by a high sensitivity immunoturbidimetric method (Beckman-Coulter).

### Statistical analysis

Normality of distributions was tested with the Kolmogorov-Smirnov test. We used Spearman's (*ρ*) test to identify correlations between variables, and curve estimation analyses when non-linear correlations were found. Statistical analyses were performed with the SPSS 17.0 statistical package. Results are presented as means (SD).

## Results

**Figure 1:**
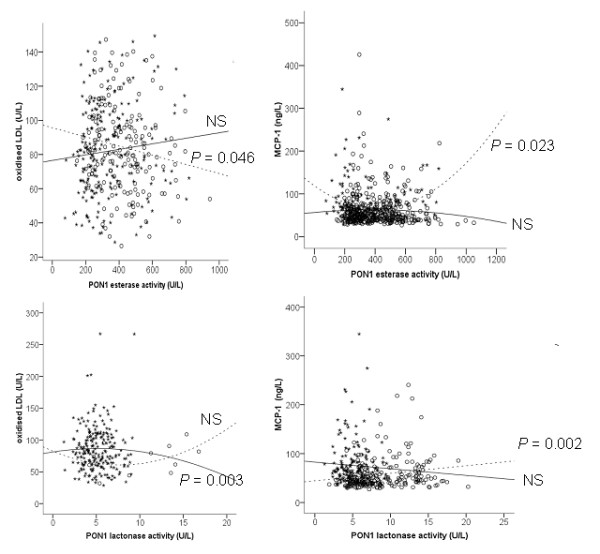
**Relationships between serum PON1 esterase and lactonase activities and oxidised LDL and MCP-1 concentrations in the control group (open circles) and HIV-infected patients (closed circles)**. The lines within the graphs represent the linear or curvilinear regression lines of the distributions of measured values in the control group (dashed lines) and the HIV-infected patients (continuous lines). NS = not significant.

Results of the selected biochemical variables are summarised in Table 1. There were significant decreases in serum PON1 activity and HDL-cholesterol and apolipoprotein A-I concentrations in HIV-infected patients with respect to the control group. Conversely, there were significant increases in plasma MCP-1 and serum CRP concentrations. Oxidised LDL levels showed a slight trend towards an increase, but differences did not reach statistical significance. We observed a significant inverse relationship between serum PON1 esterase activity and oxidised LDL levels in the control subjects (linear; *ρ *= -0.125; p = 0.046), but not in the HIV-infected patients (Figure 1A). Conversely, we observed a significant curvilinear relationship between PON1 esterase activity and MCP-1 in HIV-infected patients (p = 0.023), but not in the control group (Figure 1B). Esterase activity was also significantly correlated with HDL-cholesterol concentration in control subjects (linear; *ρ *= 0.217; p < 0.001) and in HIV-infected patients (curvilinear; p = 0.004). We did not find any statistically significant relationship between serum PON1 esterase activity and apolipoprotein A-I or CRP in the patient or control subject groups.

**Table 1:** Results of the biochemical measurements

Parameter	Control subjects	HIV-infected patients	p -value
PON1 esterase activity, U/L	410.7 (132.5)	336.8 (115.4)	< 0.001
PON1 lactonase activity, U/L	6.8 (3.0)	5.3 (1.6)	< 0.001
HDL-cholesterol, mmol/L	1.50 (0.01)	1.17 (0.03)	< 0.001
Apolipoprotein A-I, g/L	1.69 (0.03)	1.38 (0.31)	< 0.001
Oxidised LDL, U/L	85.7 (80.1)	94.0 (90.8)	NS
MCP-1, ng/L	61.54 (1.97)	71.57 (2.80)	0.003
C-reactive protein, mg/L	0.75 (0.01)	4.13 (3.30)	< 0.001

Results are shown as means and SD (in parenthesis)

We observed a significant curvilinear relationship between serum PON1 lactonase activity and oxidised LDL (p = 0.003) in HIV-infected patients (Figure 1C), but not in the control group. Conversely, lactonase activity was significantly related to MCP-1 in the control subjects (linear; *ρ *= 0.208; p = 0.002) but not in HIV-infected patients (Figure 1D). We also found significant associations between serum PON1 lactonase activity and apolipoprotein A-I (linear; *ρ *= 0.281; p < 0.001) and CRP (curvilinear; p = 0.027) in HIV-infected patients, but not in the control group.

## Discussion

The present study presents a puzzling picture in that the observed relationships between PON1 activity and several indices of oxidation and inflammation depended not only on the HIV infection but also on the substrate used to measure PON1. Methods using the non-physiological paraoxon as substrate have traditionally been used to measure PON1 activities, the levels of which have been proposed as indicators in the prediction of cardiovascular disease [1]. Recently, the lactonase activity assay using TBBL has been developed and evaluated [7,8] and, although TBBL is also a synthetic substrate, it probably measures an activity that is closer to the physiological state, since it represents a chemical reaction that is lactone-degrading and, as such, is probably closer to the physiological basis of PON1 activity. Experimental evidence suggests that, essentially, the lactonase assay measures PON1 that is tightly bound to HDL particles, while the esterase assay measures the tightly as well as the loosely bound enzyme [7]. The differences between methods, therefore, could also be related to the observed changes in structure and composition of the HDL particles during HIV infection. These changes may be due to the chronic inflammation *per se*, or to secondary effects of the antiretroviral treatments [9]. There is the possibility that these changes affect PON1 activity and that this is further influenced by the type of substrate used to measure PON1. We acknowledge that the different relationships observed between PON1 activities and MCP-1 in HIV-infected patients may, possibly, not be extrapolated to non-diseased control subjects. Nevertheless, the wide distribution of PON1 in different cell types and co-localisation with MCP-1 in most tissues [10] suggests a fundamental association between these two molecules in the regulation of oxidation and inflammation.

## Conclusions

We conclude with a cautionary note to researchers conducting clinical studies on PON1; the experimental findings can vary, as in the present study, depending on the methods of measurement used, and the disease studied. Until more specific methods using physiologically-akin substrates are developed for PON1 measurement, the simultaneous employment of at least two different substrates to measure PON1 activity would be the recommended so that the reliability of the results may be improved.

## Abbreviations

CRP: C-reactive protein; HDL: high-density lipoproteins; HIV: human immunodeficiency virus; LDL: low-density lipoproteins; MCP-1: monocyte chemoattractant protein-1; PON1: paraoxonase 1; TBBL: 5-(thiobutyl)-butyrolactone.

## Competing interests

The authors declare that they have no competing interests.

## Authors' contributions

SP, JM, JJ and JC had substantial contributions to conception and design, statistical analysis and writing the manuscript. SP, JM and CA-V recruited the subjects of the study. GA, AR, and RB-D performed the analytical measurements. All authors read and approved the final manuscript.

## References

[B1] Camps J Marsillach J Joven J The paraoxonases: role in human diseases and methodological difficulties in measurement Crit Rev Clin Lab Sci 2009 46 83 96 10.1080/10408360802610878 19255916

[B2] Alonso-Villaverde C Coll B Parra S Montero M Calvo N Tous M Joven J Masana L Atherosclerosis in patients infected with HIV is influenced by a mutant monocyte chemoattractant protein-1 allele Circulation 2004 110 2204 2209 10.1161/01.CIR.0000143835.95029.7D 15466648

[B3] Parra S Alonso-Villaverde C Coll B Ferré N Marsillach J Aragonès G Mackness M Masana L Joven J Camps J Serum paraoxonase-1 activity and concentration are influenced by human immunodeficiency virus infection Atherosclerosis 2007 194 175 81 10.1016/j.atherosclerosis.2006.07.024 16942773

[B4] Parra S Marsillach J Aragonés G Beltrán R Montero M Coll B Mackness B Mackness M Alonso-Villaverde C Joven J Camps J Paraoxonase-1 gene haplotypes are related to metabolic disturbances, atherosclerosis and immunologic outcome in HIV-infected patients J Infect Dis 2010 201 627 634 10.1086/650312 20078196

[B5] Coll B Parra S Alonso-Villaverde C Aragonès G Montero M Camps J Joven J Masana L The role of immunity in the progression of atherosclerosis in patients with HIV infection Stroke 2007 38 2477 2484 10.1161/STROKEAHA.106.479030 17673719

[B6] Eckerson HW Wyte CM La Du BN The human serum paraoxonase/arylesterase polymorphism Am J Hum Genet 1983 35 1126 1138 6316781 PMC1685985

[B7] Gaidukov L Tawfik DS The development of human sera test for HDL-bound serum PON1 and its lipolactonase activity J Lipid Res 2007 48 1637 1646 10.1194/jlr.D600045-JLR200 17435182

[B8] Marsillach J Aragonès G Beltrán R Caballeria J Pedro-Botet J Morcillo-Suárez C Navarro A Joven J Camps J The measurement of the lactonase activity of paraoxonase-1 in the clinical evaluation of patients with chronic liver impairment Clin Biochem 2009 42 91 8 10.1016/j.clinbiochem.2008.09.120 18977341

[B9] Navab M Ananthramaiah GM Reddy ST Van Lenten BJ Ansell BJ Hama S Hough G Bachini E Grijalva VR Wagner AC Shaposhnik Z Fogelman AM The double jeopardy of HDL Ann Med 2005 37 173 78 10.1080/07853890510007322 16019715

[B10] Marsillach J Mackness B Mackness M Riu F Beltrán R Joven J Camps J Immunohistochemical analysis of paraoxonases-1, 2, and 3 expression in normal mouse tissues Free Rad Biol Med 2008 45 146 157 10.1016/j.freeradbiomed.2008.03.023 18440321

